# Anlotinib plus penpulimab versus sorafenib as first-line treatment for unresectable hepatocellular carcinoma: a cost-effectiveness analysis from the perspective of the Chinese healthcare system

**DOI:** 10.3389/fonc.2026.1846223

**Published:** 2026-05-21

**Authors:** Gengliang Bai, Haoyu Yang, Weihao Ge, Ruotian Yang, Tongxiao Yu, Ran Qi

**Affiliations:** 1 School of Health Economics and Management, Nanjing University of Chinese Medicine, Nanjing, Jiangsu, China; 2 Jinling High School, Nanjing, Jiangsu, China; 3 Children’s Hospital of Nanjing Medical University, Nanjing, Jiangsu, China; 4 Department of Clinical Pharmacy, Affiliated Hospital of Jining Medical University, Jining Medical University, Jining, Shandong, China

**Keywords:** anlotinib, cost-effectiveness, penpulimab, sorafenib, unresectable hepatocellular carcinoma

## Abstract

**Background:**

In the phase III clinical trial named APOLLO (NCT04344158), anlotinib plus penpulimab significantly improved PFS and OS compared with sorafenib in the first-line treatment of unresectable hepatocellular carcinoma (uHCC). Although targeted agents combined with immunotherapy offer a new treatment option for uHCC and provide significant benefits, their cost-effectiveness in China remains unclear. This study aimed to evaluate the cost-effectiveness of anlotinib plus penpulimab for uHCC from the perspective of the Chinese healthcare system, in accordance with the latest *China Guidelines for Pharmacoeconomic Evaluations 2025*.

**Methods:**

A partitioned survival model was used to assess the cost-effectiveness of anlotinib plus penpulimab over sorafenib monotherapy as a first-line treatment therapy for uHCC from the perspective of the Chinese healthcare system. The time horizon was the patient’s lifetime, with a cycle of 21 days. Clinical information was derived from the APOLLO trial, cost and health state utility data were derived from local databases and published literature. Quality-adjusted life years (QALY) were used as the model’s main outcome indicator, sensitivity analyses were conducted.

**Results:**

In the base-case analysis, anlotinib plus penpulimab provided an additional 0.58 QALYs at an incremental total cost of United States dollar ($)24,637.23, with an incremental cost-effectiveness ratio (ICER) of $ 42,319.31/QALY, which was higher than the willingness-to-pay (WTP) threshold of $ 27,766.48/QALY. One-way sensitivity analysis showed that the results of the model were most sensitive to the utility value of the PD state and the price of penpulimab. Sensitivity analyzes indicated that our results were robust to the variation ranges of key inputs. Scenario analysis confirmed that when the prices of penpulimab was reduced by 50%, the cost-effectiveness of the anlotinib plus penpulimab is significantly improved.

**Conclusion:**

In this economic evaluation comparing two first-line treatments for patients with uHCC, anlotinib plus penpulimab was not more cost-effective than sorafenib from the perspective of the Chinese healthcare system. However, this outcome could be altered if penpulimab undergoes a substantial price reduction.

## 1 Introduction

Hepatocellular carcinoma (HCC) is the sixth most common cancer and the dominant form of liver tumor (accounting for 90% of cases) and is the leading cause of cancer-related deaths, especially in Asia and particularly in China (1–4). More than 60% of patients with HCC are diagnosed at an advanced stage without the opportunity to undergo radical surgery (5). Sorafenib has been the standard treatment for uHCC, due to its advantage of significant improvement in uHCC OS (6, 7). In recent years, the emergence of novel multi-targeted and immunosuppressive agents has altered the therapeutic landscape for a wide range of solid and hematological tumors, providing patients with promising options for treating their disease.

As a multi-targeted tyrosine kinase inhibitor, anlotinib inhibits tumor angiogenesis and growth by inhibiting the targets of vascular endothelial growth factor receptor, platelet-derived growth factor receptor, and fibroblast growth factor receptor (8, 9). The synergistic effect of anlotinib plus immunotherapy has been demonstrated in several studies, for example, in patients with non-small cell lung cancer and ovarian cancer, where combination therapy significantly improved disease control and survival benefit (10, 11). Penpulimab, the only novel differentiated PD-1 monoclonal antibody of the IgG1 isoform that is modified in the Fc segment, has been shown more effective in enhancing the efficacy of immunotherapy with fewer adverse effects (12).

The efficacy and safety of first-line treatment of advanced HCC with the combination of anlotinib and pegylated ramucirumab was initially demonstrated in the previous phase Ib/II AK105–203 study (NCT04172571) (12)in advanced HCC (13).The APOLLO study is a randomized, open, parallel-controlled, phase III trial conducted in 79 centers in China to evaluate the efficacy and safety of anlotinib plus penpulimab versus sorafenib in first-line treatment of advanced HCC. In this trial, 649 patients were enrolled, including 433 patients in the anlotinib plus penpulimab group and 216 patients in the sorafenib group. In the intention-to-treat (ITT) population, the results revealed that anlotinib plus penpulimab significantly prolonged the median PFS (6.9 months vs 2.8 months; HR = 0.52, 95%CI: 0.41-0.66, p<0.0001) and OS (16.5 months vs 13.2 months;HR=0.69, 95%Cl: 0.55-0.87, p=0.0014) in comparison with sorafenib. Compared with sorafenib, the risk of death in the anlotinib plus penpulimab group was reduced by 31% (HR = 0.69, 95%Cl: 0.55-0.87, p=0.0014). The tolerability and rationality of dose of anlotinib plus penpulimab was better. The adverse events (AEs) were similar in the anlotinib plus penpulimab and sorafenib groups. Meanwhile, compared with sorafenib, the treatment-emergent adverse events (TEAEs) were halved in the group of anlotinib plus penpulimab (16.2% vs. 29.9%), and the immune-mediated adverse events (AEs) were also lower.

In 2019, the economic burden of liver cancer in China was estimated to be US$1.11 billion (14). Considering the socioeconomic burden of HCC, the cost-effectiveness analysis of new therapies is critical for making rational decisions. Cai et al. also confirm that immune-combination-targeted (karelizumab plus apatinib) is cost-effective compared with sorafenib (15).But more cost-effective of immunotargeted therapies should be explored to strike a balance between efficacy and economic. In December 2025, the China Guidelines for Pharmacoeconomic Evaluations 2025 was released, This research is the first to utilize the APOLLO study to measure the cost-effectiveness of anlotinib plus penpulimab versus sorafenib in the first-line treatment of uHCC, in accordance with the latest *China Guidelines for Pharmacoeconomic Evaluations 2025*.

## 2 Material and methods

### 2.1 Population and interventions

The medical information of patients was derived from the phase III clinical trial named APOLLO. Participants in this study was aged between 18–75 years, with HCC diagnosed by histological or cytological examination or met the clinical diagnostic criteria according to the American Association for the Study of Liver Disease or the Standardization for Diagnosis and Treatment of Primary Hepatic Carcinoma. Patients were required to have Barcelona Clinic Liver Cancer (BCLC) stage C or be deemed unsuitable for local treatment or surgery stage B, and Child-Pugh liver function class A or B7 the baseline characteristics of the APOLLO study are shown in **Supplementary Table 1**.

A total of 649 patients were enrolled in the APOLLO study from August 2020 to June 2023, and were randomly assigned to two groups at a 2:1 ratio: (1) patients in the anlotinib plus penpulimab group received anlotinib (10 mg orally once daily, Days 1–14) plus penpulimab (200 mg intravenously on every 3 weeks); and (2) patients in the sorafenib group received sorafenib (400 mg orally twice daily). Since the APOLLO study did not report the detailed treatment regimen administered after disease progression, second-line treatment regimens were derived from a previous study that also evaluated sorafenib as first-line treatment for uHCC. Specifically, 50% of patients received bevacizumab (100 mg) plus regorafenib (40 mg), while the others received best supportive care (16).

### 2.2 Model structure

The partitioned survival model is a modeling technique used to estimate the modeling technique for estimating the survival rates and proportions of individuals at a specific point in time (17). In the partitioned survival model, the distribution of patients in three independent health states: PFS, PD and death, was obtained directly from the Kaplan-Meier curves of PFS and OS. The number of patients in the PFS state could be provided by the PFS curve, patients in the death state were determined from the remaining part of the OS curve, and patients in the PD state were the proportion between the PFS and OS curves.

**Figure 1:**
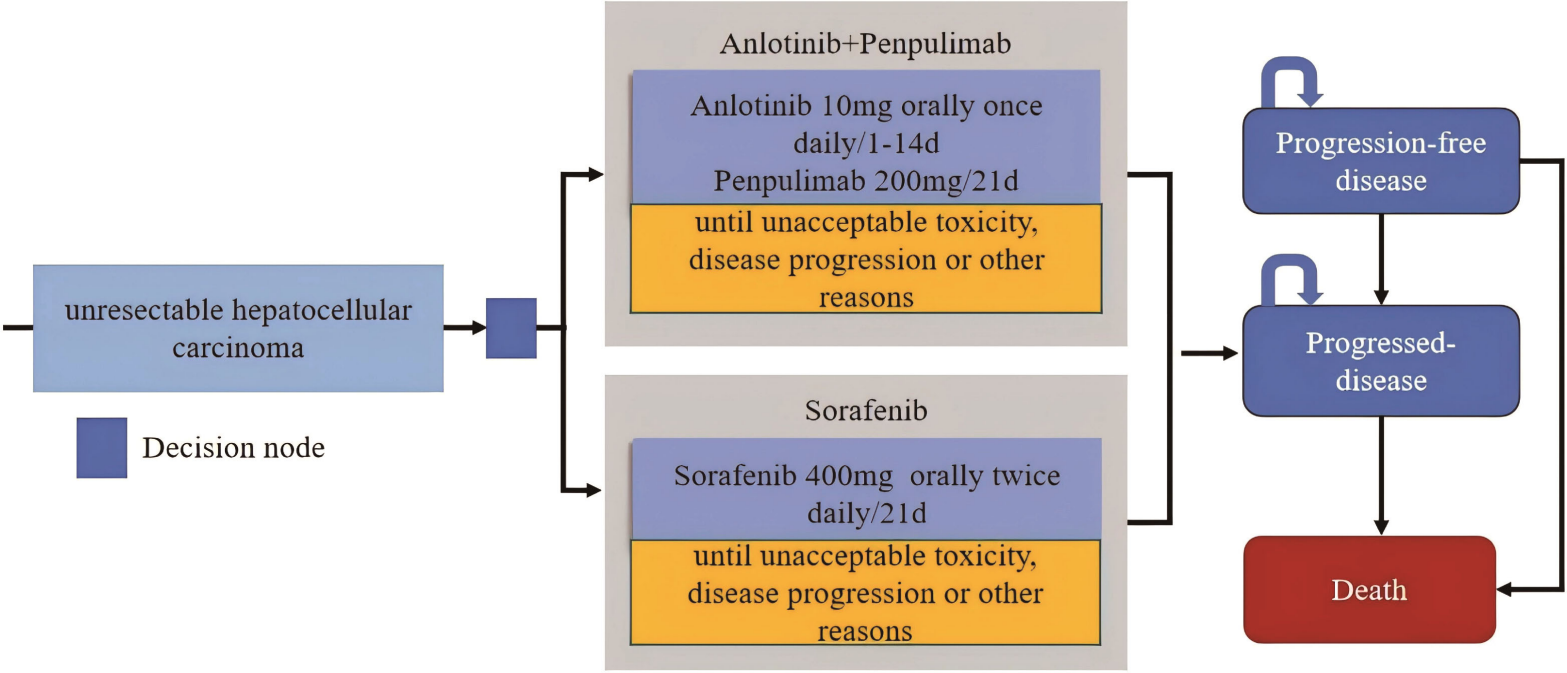
Model structure of the partitioned survival model

A partitioned survival model over lifetime horizon was constructed with 21 days cycle length (**Figure 1**). The simulation was stopped when 99% of the patients entered the death state, result in 18 years (anlotinib plus penpulimab group) vs 11 years (sorafenib group). Key outcomes included total costs, life years (LYs), QALYs, and ICERs. The model was constructed and data analysis was performed using Excel 2019.

### 2.3 Clinical data inputs

To estimate the proportion of patients in the three health states, PFS and OS curves during the APOLLO study were derived by GetData Graph Digitizer V2.20. Considering that the economic evaluation conducted in this study spanned beyond the follow-up period of the APOLLO study, six survival functions were used to fit and extrapolate PFS curves and overall survival curves, including the Exponential distribution, the Gamma distribution, the Gompertz distribution, the Weibull distribution, and the Log-logistic distribution, Log-normal distribution (18–20). The best-fitted survival functions were selected according to visual assessment, Akaike information criterion, Bayesian information criterion and oncologist’s opinions. Finally, the PFS distribution and OS distribution were Log-normal and Log-logistic for the anlotinib plus penpulimab group and Log-logistic and Log-normal for the sorafenib group, respectively (18).The proportion of patients was calculated using the selected optimal survival function. The AIC and BIC values of the optimal survival function and Kaplan-Meier fitting curves are shown in **Supplementary Table 2**, and the optimal fitting distributions and parameters are presented in **Supplementary Table 3**.

### 2.4 Cost and utility data

Only direct healthcare costs were included in the analysis, including drug costs, hospitalization costs, administration costs, subsequent therapy costs, AEs management costs, and best supportive care costs. Drug costs were calculated based on the drugs and dosages used in the clinical trials, drug prices were derived based on the median of the median bid prices by province from the Pharmaceutical Intelligence Database (https://db.yaozh.com/). The unit prices and cycle costs for sorafenib, bevacizumab, and regorafenib based on the 2026 winning bid prices from the Yaozhi.com database and the results of China’s 10th National Centralized Volume-Based Procurement. The detailed process is as follows: In the 2026 Yaozhi.com database, the median price of sorafenib 200 mg is $1.038. According to APOLLO, the dosage regimen for sorafenib is 400 mg twice daily for 21 days; therefore, the cycle cost of sorafenib is $87.173; The median price of bevacizumab 100 mg in the 2026 Yaozhi.com database was $138.742. According to the bevacizumab prescribing information, the dosage is 15 mg/kg. In this study, we assume the standard body weight of Chinese patients is 60 kg, which is the standard body weight recommended for innovative drugs when accessing the National Reimbursement Drug List (NRDL) in China; therefore, the cycle cost of bevacizumab is $1,248.677; Since regorafenib has been included in China’s national centralized bulk procurement, we use the highest price from the procurement list, which is $0.795 per tablet (40mg). According to the regorafenib prescribing information (160 mg once daily for 21 days), the cycle cost of regorafenib is $66.822. Other costs, such as subsequent therapy, hospitalization, AEs management, and supportive care costs, were obtained from the relevant published literature (21–23). In the sensitivity analysis, we performed uncertainty analyzes for each cost variable to assess its impact on the results. All cost inputs were reported as 2025 US dollars with Chinese yuan transformed to US dollars by the exchange rate in: US$1 = ¥7.1788.

Each health state was assigned a health utility preference on a scale of 0 (death) to 1 (perfect health). Given that utility values were not reported in the APOLLO study, we calculated them by leveraging the previously reported utility values with similar features in advanced HCC. Based on previously published cost-effectiveness analyzes, the utility values of PFS state and the PD state were 0.76 and 0.68, respectively (24). It was assumed that all AEs occurred in the first cycle. However, this assumption is subject to certain limitations, as it fails to enable accurate estimation of the corresponding resource consumption as well as the survival benefit reduction attributable to adverse events. The cost and utility value parameters and their ranges are presented in **Table 1**. The WTP threshold was set at two times China’s 2025 per capita Gross Domestic Product (GDP) ($27,766.48/QALY).

**Table 1:** Key model parameters.

Parameters	Base-case value	Ranges	Distribution	Source
Cost ($)				
Anlotinib (10mg)	34.347	27.478-41.216	Gamma	yaozh database
Penpulimab (100mg)	497.298	397.838-596.758	Gamma	yaozh database
Sorafenib (200mg)	1.038	0.830-1.246	Gamma	yaozh database
Regorafenib (40mg)	0.795	0.636-0.954	Gamma	yaozh database
Bevacizumab(100mg)	138.742	110.993-166.490	Gamma	yaozh database
Supportive care(/cycle)	174.679	139.743-209.614	Gamma	[ (21)]
Routine follow-up per unit ($)				
Enhanced CT	57.126	45.990-68.980	Gamma	[ (22)]
Bed	6.482	5.220-7.830	Gamma	[ (22)]
Diagnosis	3.084	1.550-4.660	Gamma	[ (22)]
Intravenous injection	1.699	1.550-2.140	Gamma	[ (22)]
Care	3.702	2.980-4.470	Gamma	[ (22)]
blood biochemical examination	46.206	37.200-55.800	Gamma	[ (22)]
blood test	3.084	2.490-3.730	Gamma	[ (22)]
urinalysis	0.619	0.500-0.750	Gamma	[ (22)]
Management of adverse event ($)				
Hypertension	34.824	28.360-42.550	Gamma	[ (23)]
Platelet count decreased	1496.480	1240.170-1771.670	Gamma	[ (22)]
AST	55.526	45.230-67.840	Gamma	[ (23)]
White blood cell count decreased	114.282	51.110-357.800	Gamma	[ (22)]
Neutrophil count decreased	114.282	51.110-357.800	Gamma	[ (22)]
Lymphocyte count decreased	463.600	377.650-566.480	Gamma	[ (21)]
increased blood bilirubin	111.493	90.820-136.240	Gamma	[ (23)]
Utility values				
PFS	0.76	0.61-0.91	Beta	[ (24)]
PD	0.68	0.54-0.82	Beta	[ (24)]
Incidence of adverse events				
Hypertension_ANLO	0.17	0.153-0.187	Beta	[ (13)]
Platelet count decreased_ANLO	0.08	0.072-0.088	Beta	[ (13)]
AST_ANLO	0.04	0.036-0.044	Beta	[ (13)]
White blood cell count decreased_ANLO	0.05	0.045-0.055	Beta	[ (13)]
Neutrophil count decreased_ANLO	0.06	0.054-0.066	Beta	[ (13)]
Lymphocyte count decreased_ANLO	0.04	0.036-0.044	Beta	[ (13)]
increased blood bilirubin_ANLO	0.04	0.036-0.044	Beta	[ (13)]
Hypertension_SORA	0.1	0.09-0.11	Beta	[ (13)]
Platelet count decreased_SORA	0.06	0.054-0.066	Beta	[ (13)]
AST_SORA	0.06	0.054-0.066	Beta	[ (13)]
Others				
Discount rate	4.5%	0%-8%	Beta	[ (16)]

### 2.5 Sensitivity and scenario analysis

To examine the robustness of the results of the model-based analyzes, a one-way sensitivity analysis and a probabilistic sensitivity analysis (PSA) were carried out in this study. In the one-way sensitivity analysis, drug prices were taken to be ±20% of their baseline values, and the range of cost and utility value data was determined based on either the reported 95% CI in the referenced studies or by assuming a 20% variation from the base-case value. The discount rate fluctuated in the range of 0%-8%. The results of one-way sensitivity analysis are presented as tornado plot. In the PSA, a Monte Carlo simulation with 1,000 iterations was generated by simultaneously sampling the key model parameters from the predefined distributions. The gamma distribution was used for the cost parameters and the beta distribution for the utility value and adverse effect rate parameters (25). Cost-effectiveness acceptability curves and scatter plots were used to evaluate the cost-effectiveness of the anlotinib plus penpulimab versus sorafenib across different willingness-to-pay thresholds. We then conducted a scenario analysis with respect to five conditions: 25% and 50% price reductions of penpulimab; and 25% and 50% price reductions of anlotinib.

## 3 Results

### 3.1 Base-case analysis

Compared with sorafenib, anlotinib plus penpulimab provided an additional benefit of 0.582 QALYs with an incremental cost of $24,637.228 for patients with uHCC (**Table 2**). The ICERs of anlotinib plus penpulimab compared to sorafenib was 42,319.309$/QALY. The results suggested that anlotinib plus penpulimab was not a cost-effective option compared with sorafenib at the WTP threshold of $27,766.48/QALY.

**Table 2:** Base-case analysis results.

Group	Cost/$	QALYs	Incremental cost/$	Incremental QALYs	ICER/($/QALY)
Anlotinib+Penpulimab	37,381.003	1.502	24,637.228	0.582	42,319.309
Sorafenib	12,743.775	0.920	NA	NA	NA

### 3.2 Sensitivity analysis results

**Figure 2:**
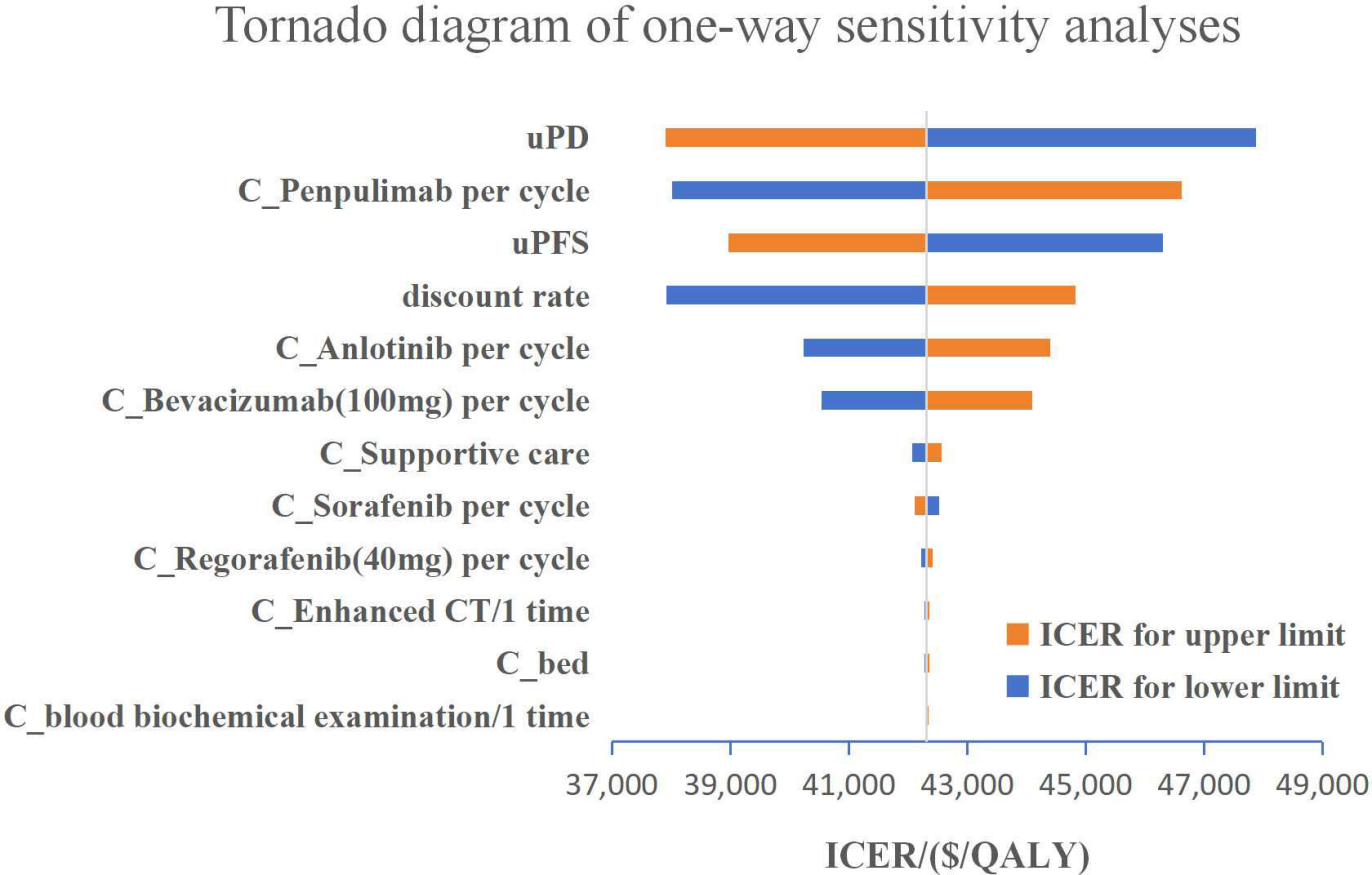
Tornado diagram of one-way sensitivity analyses.

**Figure 3:**
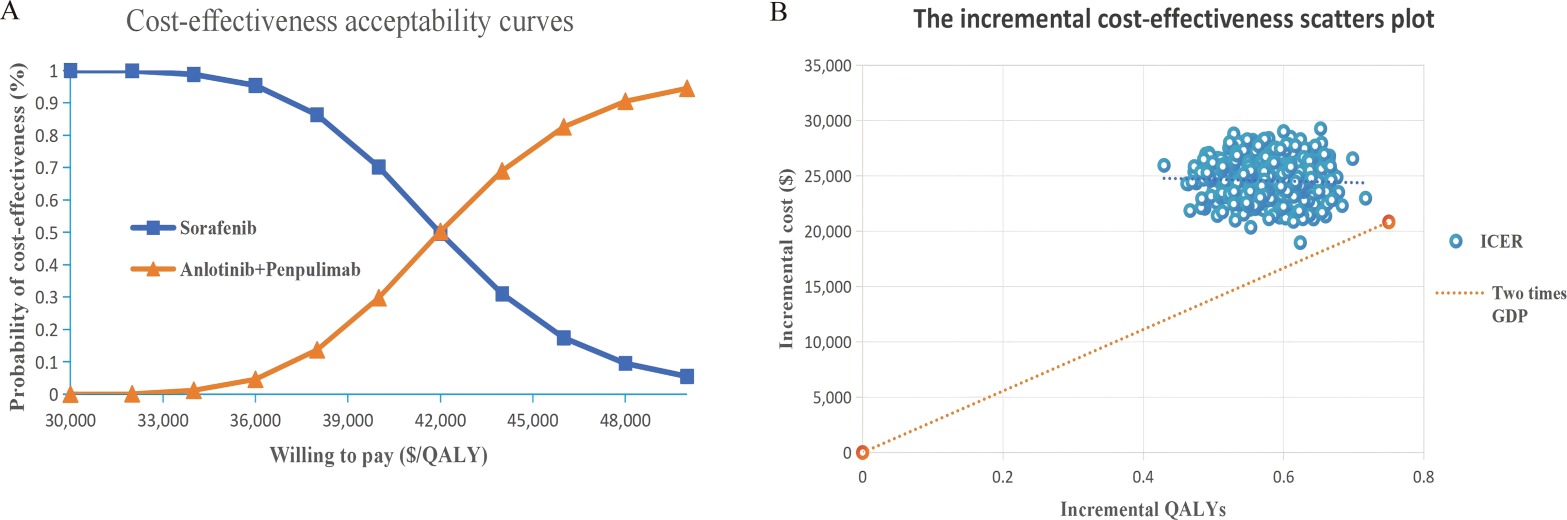
**(A)** Probabilistic sensitivity analyses-Cost-effectiveness acceptability curves. **(B)** Probabilistic sensitivity analyses-Scatter Plot. QALY Quality-adjusted life year, ICER Incremental cost-effectiveness ratio, WTP=$27,766.48 the value of two times the per capita GDP.

One-way sensitivity analysis showed that the results of the model were most sensitive to the utility value of the PD state and the price of penpulimab. Other considerable influential parameters were the utility value of the PFS state, discount rate, price of anlotinib, and price of bevacizumab (**Figure 2**). In the PSA, the scatter plots showed that the cost-effectiveness probability of anlotinib plus penpulimab was 0% under the preset threshold in China (**Figure 3A**). The cost-effectiveness acceptability curve showed that the probability of cost-effectiveness of the anlotinib plus penpulimab under the willingness-to-pay threshold of two times the per capita GDP in China was 0%, but if we set the WTP threshold at $42,000/QALY, anlotinib plus penpulimab would have a chance of 50.3% to be a cost-effective option, and when the WTP threshold is raised to $50,000/QALY, this probability would increases to 95% (**Figure 3B**).

### 3.3 Scenario analysis results

**Figure 4:**
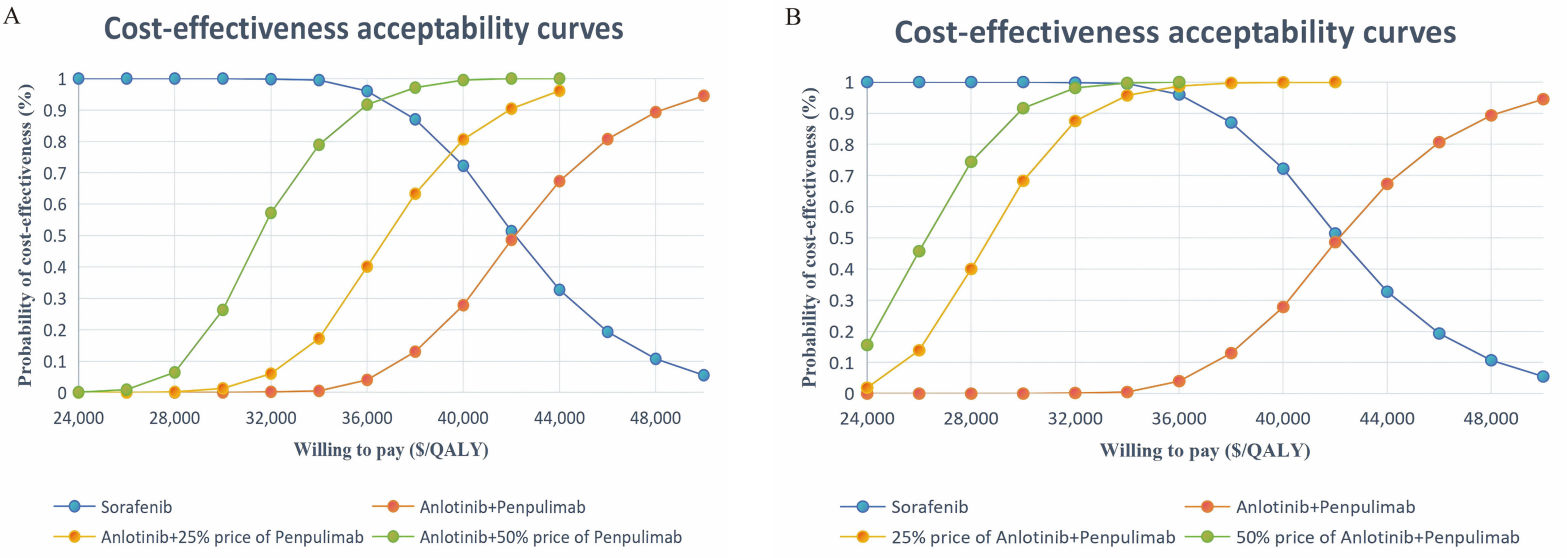
Scenario analysis of Acceptability curves of 25% and 50% reduction in the price of penpulimab **(A)** and anlotinib **(B)** from the base-case value. QALY Quality-adjusted life year.

In Scenarios 1 and 2 (**Figure 4A**), for a 25% and 50% reduction in the price of penpulimab from the base-case value, respectively, the ICER values of anlotinib plus penpulimab compared with sorafenib were $36,941.75 and $31,564.19, corresponding to probabilities of being cost-effective of 32.70% and 77.60%, respectively. In Scenarios 3 and 4 (**Figure 4B**), where the price of anlotinib was reduced by 25% and 50%, respectively, from the base-case value, the ICERs for anlotinib plus penpulimab compared to sorafenib were $28,964.31 and $26,364.44, corresponding to probabilities of being cost-effective of 84.40% and 92.90%. The detailed results are presented in **Table 3**.

**Table 3:** Scenario analysis results.

Group	Cost/$	QALYs	Incremental cost/$	Incremental QALYs	ICER/($/QALY)
Scenario 1 (25% price reduction of Penpulimab)					
Anlotinib+Penpulimab	34,250.323	1.502	21,506.548	0.582	36,941.748
Sorafenib	12,743.775	0.920	NA	NA	NA
Scenario 2 (50% price reduction of Penpulimab)					
Anlotinib+Penpulimab	31,119.643	1.502	18,375.868	0.582	31,564.186
Sorafenib	12,743.775	0.920	NA	NA	NA
Scenario 3 (25% price reduction of Anlotinib)					
Anlotinib+Penpulimab	29,606.064	1.502	16,862.288	0.582	28,964.314
Sorafenib	12,743.775	0.920	NA	NA	NA
Scenario 4 (50% price reduction of Anlotinib)					
Anlotinib+Penpulimab	28,092.484	1.502	15,348.709	0.582	26,364.442
Sorafenib	12,743.775	0.920	NA	NA	NA

## 4 Discussion

The efficacy and safety of anlotinib plus penpulimab as the first-line treatment option for uHCC have been confirmed in the APOLLO study, and it is expected to become the preferred treatment option for uHCC patients. However, while new treatment options bring significant benefits, they also lead to a significant increase in the consumption of medical resources, which inevitably raises concerns among government policymakers and medical practitioners. As we all know, this study is the first to evaluate the cost-effectiveness of anlotinib plus penpulimab compared with sorafenib as a first-line treatment for uHCC based on the latest efficacy and safety data released by the APOLLO study in accordance with the latest *China Guidelines for Pharmacoeconomic Evaluations 2025*, and its results are of great guiding significance for medical insurance policy makers and medical practitioners.

The results showed that anlotinib plus penpulimab could obtain an additional 0.582 QALY compared with sorafenib monotherapy. Meanwhile, the treatment cost increased by $24,637.228, and the ICER was 42,319.309$/QALY, above the WTP threshold. In the one-way sensitivity analysis, health utility values, the cycle cost of treatment drugs, and the discount rate were key influencing factors. In the PSA, the probability of anlotinib plus penpulimab being cost-effective was 0%, similar to the findings of Luo et al (26). But Luo et al. used a 10-year modeling horizon, this study employs a lifetime modeling horizon, which better captures the clinical course of uHCC patients and thus allows for a more accurate calculation of survival benefits and healthcare resource utilization for uHCC patients. Meanwhile, the Chinese Guidelines on Pharmacoeconomics have been updated to the 2025 edition. Regarding the discount rate and WTP threshold, this study employs the latest discount rate (4.5%) and a WTP threshold of twice the 2025 per capita GDP, enabling more precise calculation of study results and assessment of the probability that anlotinib plus penpulimab is cost-effective. This study incorporates the costs of all AEs, which provides greater precision in both total cost calculations and health benefit assessments. In order to fully assess its cost-effectiveness, this study conducted a scenario analysis of the price reduction of the main therapeutic drugs (anlotinib and penpulimab) to explore at what price the two drugs would be cost-effective. The results showed that the probability of cost-effectiveness of the combination therapy increased significantly when penpulimab were reduced by 50%.

Multiple studies have confirmed that combination therapy significantly prolongs the survival of uHCC and is superior to monotherapy or targeted therapy (27, 28). The mainstream combination strategies include: immune checkpoint inhibitor plus anti-angiogenic drug, immune checkpoint inhibitor plus targeted drug and dual immune checkpoint inhibitor (27). Some studies have been carried out to find out whether the combined therapies are cost-effective. The study conducted by Cheng et al. proved that the dual-immune therapy (toripalimab plus durvalumab) has both survival benefits and economic feasibility in uHCC (24). However, this study explored the cost-effectiveness of the dual immunotherapy therapy from the perspective of the US health system. Liu et al. conducted a study by constructing a partitioned survival model to compare the cost-effectiveness of atezolizumab plus bevacizumab versus traditional treatment with sorafenib monotherapy for first-line treatment of uHCC in the US and Chinese healthcare systems (29). The results showed that atezolizumab plus bevacizumab was unlikely to be a cost-effective option for the treatment of uHCC compared with the traditional sorafenib monotherapy therapy in the context of the US and Chinese healthcare systems.

The results of Cai et al. evaluated the cost-effectiveness of camrelizumab plus apatinib as a first-line treatment for advanced HCC in the Chinese healthcare system based on the CARES-310 trial (15). The results showed that compared with the sorafenib monotherapy therapy, the ICER of the immune checkpoint inhibitor plus targeted drugs (camrelizumab plus apatinib) was 9,147.012$/QALY, which became a cost-effective treatment therapy for first-line advanced hepatocellular carcinoma in China under the background of $39,855.79/QALY as the WTP threshold. We also conducted a cost-effectiveness analysis of immune checkpoint inhibitors plus anti-angiogenic drugs (penpulimab plus anlotinib). Different from Cai et al, we found that immune checkpoint inhibitors plus anti-angiogenic drugs were not cost-effective in the treatment of uHCC based on the APOLLO study, and this difference in trial selection also highlighted the differences between the two studies. Therefore, whether immune checkpoint inhibitors plus anti-angiogenic drugs are cost-effective in the treatment of uHCC needs further research to explore, and there is still a lack of economic research from the perspective of the Chinese healthcare system. It is expected that more scholars will evaluate the economics of the dual immunotherapy therapy in the treatment of uHCC from the Chinese perspective in the future.

In the current treatment of uHCC, it is urgent to seek first-line treatment options with prominent survival benefits and cost-effectiveness. The high investment in the research and development of new drugs has led to the high cost of immune checkpoint inhibitors, which are usually not well covered by the government medical insurance system. Since 2017, The adjustment of the NRDL in China has become regularized, and more innovative drugs have been included in the NRDL timely. This study found that probability of cost-effectiveness of the combination therapy increased significantly when penpulimab were reduced by 50%. Against the background of reform of the medical insurance payment system and the annual adjustment of the NRDL in China, if a price reduction is implemented for penpulimab, we suggest that decision-makers may consider the survival benefits of high-value drugs for cancer patients, so as to appropriately allocate more medical resources to this group. As explored in the scenario analysis of this study, in the future, the prices of high-value drugs could be lowered through NRDL negotiations. This would enable the treatment schemes that offer substantial survival benefits to better serve the public.

In addition to the price reduction of high-value drugs, another way to improve cost-effectiveness results is to select patients that are more responsive to immunotherapy. It has been confirmed that HCC is a heterogeneous tumor with different immune responses and tumor biology (30), our study showed that anlotinib plus penpulimab was cost-effective in the initial treatment of uHCC, when penpulimab were reduced by 50%. The results provided valuable reference for policy makers and medical practitioners in participating in uHCC management and helped to optimize the allocation of medical resources and decision-making process.

There are several limitations to this study. First, the use of parametric distribution fitting to obtain health benefits and costs beyond the follow-up period will introduce some uncertainty into the study. Fortunately, we conducted uncertainty analysis on the results of the basic analysis to explore its robustness. Secondly, the model in this study is based on the data of APOLLO study, which is a randomized, double-blind clinical study and cannot fully reflect the real clinical diagnosis, treatment and resource consumption of uHCC patients. Third, the subsequent therapy was not clarified in the APOLLO study. This study selected regorafenib and bevacizumab as the subsequent therapy referred to a cost-effectiveness analysis of uHCC. However, it should be noted that other targeted drugs and anti-angiogenic drugs can also be used as subsequent therapy. This study features a rigorous design and has conducted sufficient uncertainty analysis and scenario analysis. Therefore, this study still holds high value.

## 5 Conclusion

The current cost-effectiveness analysis shows that anlotinib plus penpulimab is not a cost-effective option for first-line treatment of uHCC patients compared with sorafenib monotherapy in the perspective of Chinese healthcare system. But scenario analysis found that the reductions in the prices of penpulimab can significantly improve the cost-effectiveness of the combination therapy.

## Data Availability

The original contributions presented in the study are included in the article/**Supplementary Material**. Further inquiries can be directed to the corresponding authors.

## Supplementary material

Supplementary Table 1 Baseline characteristics between the Anlotinib+penpulimab and Sorafenib groups.

Supplementary Table 2 Summary of statistical goodness-of-fit of Kaplan-Meier curves in APOLLO trial.

Supplementary Table 3 Survival parameters for the two treatment strategies.

Supplementary Figure 1 Raw Kaplan-Meier curves and best-fit curves for OS and PFS in the Anlotinib+penpulimab/Sorafenib group.
