# Supplementary material for: Anlotinib plus penpulimab versus sorafenib as first-line treatment for unresectable hepatocellular carcinoma: a cost-effectiveness analysis from the perspective of the Chinese healthcare system
Source: Front Oncol. 2026 May 21;16:1846223. doi: 10.3389/fonc.2026.1846223 (PMC13233339; doi:10.3389/fonc.2026.1846223)
Supplement: Supplementary Table 1 — Baseline characteristics between the Anlotinib+penpulimab and Sorafenib groups. [file DataSheet1.docx]

Supplementary Material

# Supplementary Figures and Tables

## Supplementary Figures

**
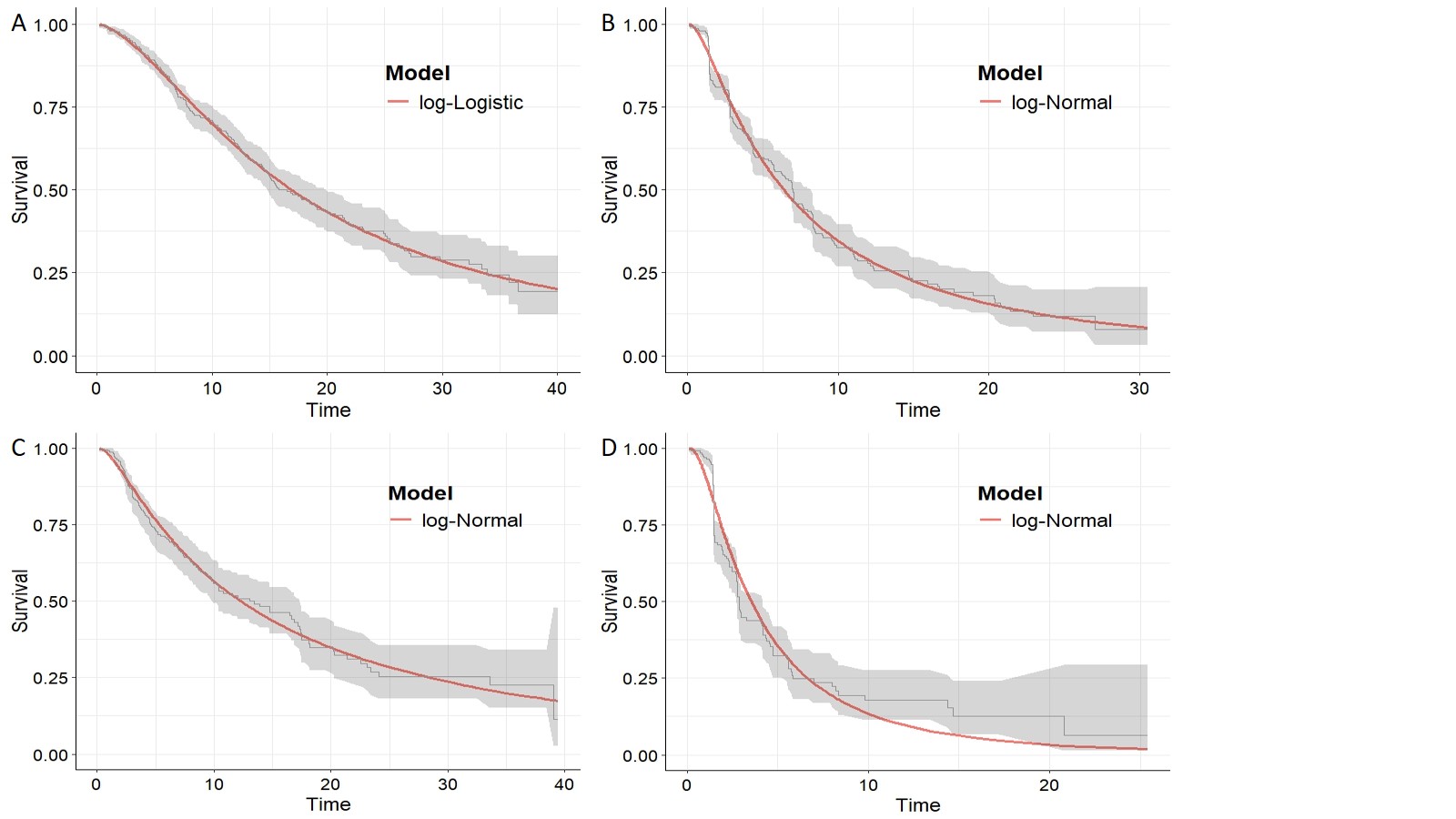
**

**Supplementary Figure 1.** Raw K-M curves and best-fit curves for OS and PFS in the Anlotinib+penpulimab/Sorafenib group. A and B represent OS and PFS in the Anlotinib+penpulimab group, respectively. C and D represent OS and PFS in the Sorafenib group, respectively.

## Supplementary Figures

- **Supplementary Table 1**. Baseline characteristics between the Anlotinib+penpulimab and Sorafenib groups

|  | Anlotinib+penpulimab（n=433） | Sorafenib  （n=216） |
| --- | --- | --- |
| **Age, years** | 57.0 (50.0–65.0) | 56.0 (50.0–65.0) |
| **Age group** |  |  |
| ≥65 years | 118（27%） | 161（75%） |
| <65 years | 315（73%） | 55（25%） |
| **Sex** |  |  |
| Male | 371（86%） | 180（83%） |
| Female | 62（14%） | 36（17%） |
| **Ethnicity** |  |  |
| Chinese | 433（100%） | 216（100%） |
| **ECOG performance status** |  |  |
| 0 | 247（57%） | 122（56%） |
| 1 | 186（43%） | 94（44%） |
| **Barcelona Clinic Liver Cancer stage*** |  |  |
| B | 79/432（18%） | 42（19%） |
| C | 353/432（82%） | 174（81%） |
| **Child-Pugh score*** |  |  |
| A | 399/432 (92%) | 201 (93%) |
| B | 33/432 (8%) | 15 (7%) |
| **Disease aetiology** |  |  |
| Hepatitis B virus positive | 365 (84%) | 181 (84%) |
| Hepatitis C virus positive | 16 (4%) | 7 (3%) |
| **Macrovascular invasion or extrahepatic metastasis** | 348 (80%) | 173 (80%) |
| Macrovascular invasion | 179 (41%) | 87 (40%) |
| Extrahepatic metastasis | 267 (62%) | 137 (63%) |
| Both | 98 (23%) | 51 (24%) |

Abbreviations: *Barcelona Clinic Liver Cancer stage，Its core value lies in integrating tumor burden, liver function reserve, and the patient's physical status；Child-Pugh score, The Child-Pugh Score is one of the "gold standards" in the clinical assessment of liver function reserve.

- **Supplementary Table 2.** Summary of statistical goodness-of-fit of KM curves in APOLLO trial

|  | Exponential | Gamma | Gompertz | Weibull | Log-logistic | Log-normal |
| --- | --- | --- | --- | --- | --- | --- |
| OS curve of the Anlotinib+penpulimab group |  |  |  |  |  |  |
| AIC | 1833.761 | 1816.390 | 1830.745 | 1819.224 | **1812.341** | 1823.074 |
| BIC | 1837.832 | 1824.531 | 1838.886 | 1827.366 | **1820.482** | 1831.216 |
| PFS curve of the Anlotinib+penpulimab group |  |  |  |  |  |  |
| AIC | 1351.660 | 1344.988 | 1353.322 | 1349.238 | 1328.001 | **1323.522** |
| BIC | 1355.710 | 1353.087 | 1361.422 | 1357.338 | 1336.101 | **1331.622** |
| OS curve of the Sorafenib group |  |  |  |  |  |  |
| AIC | 958.699 | 959.348 | 959.096 | 960.355 | 949.970 | **945.941** |
| BIC | 962.075 | 966.099 | 965.846 | 967.105 | 956.720 | **952.691** |
| PFS curve of the Sorafenib group |  |  |  |  |  |  |
| AIC | 634.255 | 625.703 | 633.600 | 632.506 | 595.080 | **593.873** |
| BIC | 637.612 | 632.417 | 640.313 | 639.219 | 601.793 | **600.587** |

Abbreviations: AIC, Akaike information criterion; BIC, Bayesian information criterion; OS, overall survival; K-M: Kaplan-Meier; PFS, progression-free survival;

- **Supplementary Table 3.** Survival parameters for the two treatment strategies

| **regimens** | **K-M curve** | **model** | **Survival parameters** |
| --- | --- | --- | --- |
| Anlotinib+penpulimab group | OS | Log-logisticl | Scale=0.011 Shape=1.603 |
| Anlotinib+penpulimabgroup | PFS | Log-normal | Meanlog=1.858 Sdlog=1.126 |
| Sorafenib group | OS | Log-normal | Meanlog=2.508 Sdlog=1.241 |
| Sorafenib group | PFS | Log-normal | Meanlog=1.263 Sdlog=0.938 |

Abbreviations:OS, overall survival; K-M: Kaplan-Meier; PFS, progression-free survival
